# Better care for children with appendicitis: implementation of antibiotic stewardship optimizes postoperative therapy

**DOI:** 10.3205/dgkh000535

**Published:** 2025-03-05

**Authors:** Sebastian Beltz, Stephanie Fischer, Frank Huenger, Reza Vahdad, Hermann Kalhoff, Andreas Leutner

**Affiliations:** 1 Department of Pediatric Surgery and Pediatric Urology, Children’s Hospital, Klinikum Dortmund, Germany; 2 University Witten/Herdecke, Witten, Germany; 3 Department of Pharmacology, Klinikum Dortmund, Germany; 4 Institute for Hospital Hygiene and Clinical Microbiology, Klinikum Dortmund, Germany; 5 Department of Pediatric Surgery and Pediatric Urology, University Hospitals of Gießen and Marburg, Germany; 6 Department of Pediatrics and Adolescent Medicine, Children’s Hospital, Klinikum Dortmund, Germany

**Keywords:** appendicitis children, postoperative treatment, antibiotic stewardship, pediatric surgery, improved outcome

## Abstract

**Aim:**

Appendectomy is the most frequent emergency abdominal operation in children, who regularly present with complicated acute appendicitis and thus need targeted antibiotic therapy. While in other fields antibiotic stewardship (ABS) is becoming increasingly well established, these principles are not consistently followed in pediatric surgery. With this study, we aimed to analyze the effects of the implementation of an ABS-compliant SOP on the postoperative care of patients.

**Material and methods:**

We compared the quality of antibiotic therapy before and after the implementation of standard operating procedure (SOP) for the peri-/postoperative antibiotic treatment of appendicitis in 2020. Pediatric patients who had undergone appendectomy were evaluated based on an algorithm presenting recommended antibiotic therapy of appendicitis, according to the current literature and good clinical practice. 165 patients were evaluated before and 209 patients after the implementation of SOP.

**Results:**

The mean number of cases in which antibiotic therapy was given postoperatively was 10.5% lower (p-value 0.036) and the median quality-of-treatment score increased by 31.2% (p<0.0001) after the implementation of the SOP. The median length of antibiotic treatment in cases of advanced-stage appendicitis was 2.0 days shorter (p=0.062). The rate of oral antibiotic treatment after discharge decreased by 25.6% (p<0.0001). We observed no significant effects on the median length of hospital stay or the complication rate.

**Conclusion:**

The implementation of SOP based on the principles of ABS positively influenced the quality of treatment after pediatric appendectomy. The algorithm developed in this study may help pediatric surgeons to improve their antimicrobial assessment.

## Zusammenfassung

**Ziel:**

Die Entfernung des Wurmfortsatzes ist die häufigste abdominelle Notfalloperation bei Kindern, die verhältnismäßig oft mit einer fortgeschrittenen Appendizitis vorstellig werden und daher eine zielgerichtete antibiotische Therapie benötigen. Während in anderen medizinischen Fachbereichen die Prinzipien des Antibiotic Stewardship (ABS) zunehmend etabliert sind, werden diese in der Kinderchirurgie immer noch nicht konsequent berücksichtigt. Ziel dieser Studie war es daher, die Auswirkungen der Implementierung einer ABS-konformen SOP auf die postoperative Versorgung dieser Patientengruppe zu untersuchen.

**Material und Methoden:**

Wir verglichen die Qualität der peri- und postoperativen Antibiotikatherapie vor und nach der Implementierung einer Standardarbeitsanweisung (SOP) im Jahr 2020. Die Versorgung pädiatrischer Patienten, bei denen eine Appendektomie durchgeführt wurde, wurde anhand eines Algorithmus bewertet, der aus unserer Sicht die empfohlene Antibiotikatherapie gemäß aktueller Literatur und guter klinischer Praxis darstellt. Insgesamt wurden 165 Patientenfälle vor und 209 Patienten nach Einführung der SOP ausgewertet.

**Ergebnisse:**

Nach Einführung der SOP wurde in durchschnittlich 10,5% weniger Fällen eine postoperative antibiotische Therapie initiiert (p-Wert 0,036). Die nach dem in diesem Rahmen entwickelten Score evaluierte Qualität der Behandlung verbesserte sich um 31,2% (p<0,0001). Die mediane Dauer der Antibiotikabehandlung bei fortgeschrittener Appendizitis war 2,0 Tage kürzer (p=0,062). Der Anteil der fortgeführten oralen Antibiotikabehandlung nach Entlassung sank um 25,6% (p<0,0001). Dabei wurde kein signifikanter Effekt auf die mediane Krankenhausverweildauer oder die Komplikationsrate beobachtet.

**Schlussfolgerung:**

Die Implementierung einer auf den Prinzipien des ABS basierenden SOP hatte einen positiven Einfluss auf die Behandlungsqualität nach Appendektomie im Kindesalter. Der für diese Studie entwickelte Behandlungsalgorithmus kann zukünftigen Kinderchirurgen als Entscheidungshilfe für die Wahl der korrekten antimikrobiellen Therapie bei Appendizitis dienen.

## Introduction

Acute appendicitis (AA), with a lifetime risk of 7–8% and a peak incidence in the early teens, is the most common reason for emergency treatment and surgery in pediatric patients with acute abdominal pain [1], [2]. Not only the incidence, but also the severity upon presentation at the surgical unit is higher in the population of low- and middle-income countries as well as in the younger population worldwide [3], [4]. While the proportion of complicated acute appendicitis (CAA) in adult patients is estimated to be around 30%, literature shows a much higher proportion of advanced disease, perforation and/or abscess formation in the pediatric population, for various reasons [3], [5], [6]. 

In the absence of a precise definition of what characterizes CAA as opposed to non-complicated appendicitis (NCA), a common basis for comparison is still lacking [7]. Nevertheless, cases of CAA or even organ perforation in children certainly require careful intraoperative assessment and postoperative care, including effective antibiotic treatment, as they are associated with an increased likelihood of postoperative complications such as surgical site infection (SSI) or abscess formation. To prevent these complications, optimal antibiotic therapy after appendectomy has been the subject of scientific attention for years, but a consistent approach has yet to be found. The principles of antibiotic stewardship (ABS), which aim to standardize and improve anti-inflammatory treatment, have been widely implemented in adult and pediatric medicine [8], [9], [10], [11]. However, several publications indicate a lack of knowledge about the aspects of anti-inflammatory treatment and prophylaxis within the pediatric surgical community [12], [13], [14], [15]. 

Certainly, it is indisputable that young patients suffering from appendicitis at all stages deserve optimal treatment based on professional microbiological assessment in the context of intraoperative findings, expected pathogens and their possible antimicrobial resistance. Therefore, we aimed to optimize our clinical approach to postoperative antibiotic management by introducing a standardized pathway for all stages of appendicitis. By combining Good Clinical Practice (GCP) with the principles of ABS, we hypothesized that we could regulate antibiotic use more efficiently to improve postoperative management of children.

**Figure 1:**
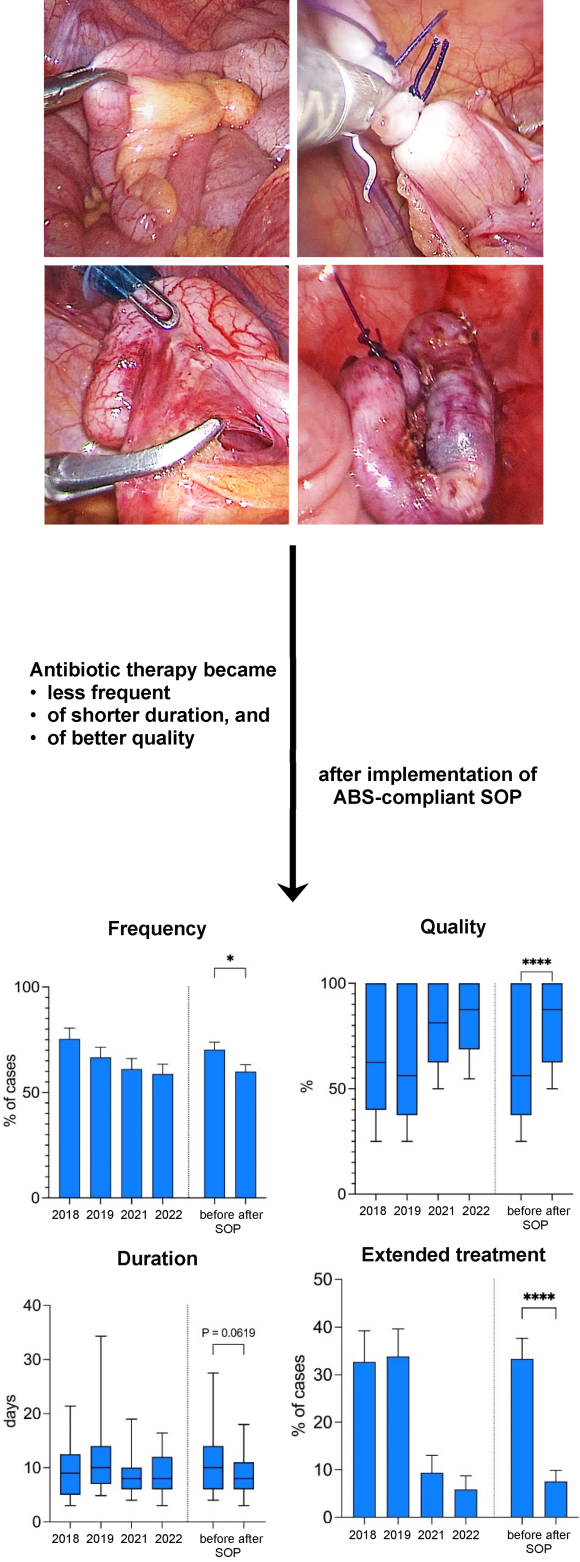
Optimizing postoperative treatment of pediatric appendicitis with ABS-compliant SOP; a single-center retrospectove analysis of the impact of the implementation of an antibiotic stewardship (ABS)-compliant standard operating procedure (SOP) on the quality of postoperative antibiotic treatment in 386 children undergoing appendectomy.

Figure 1 (Fig. 1) provides a graphical abstract of the results obtained in this manner.

## Material and methods

In the summer of 2020, we implemented a standard operating procedure (SOP) for peri/postoperative antibiotic regimens based on intraoperative findings: Patients with non-complicated appendicitis (NCA) should not receive postoperative antibiotic therapy in addition to the mandatory preoperative single-shot Cefuroxime. Patients with complicated acute appendicitis (CAA) were to receive postoperative Cefuroxime/Metronidazole, and in cases of perforation or intra-abdominal abscess (IAA), Piperacillin/Tazobactam was demanded to cover expected pathogens [16], [17], [18], [19], [20]. NCA was defined by the absence of peritonitis, ulceration, and gangrenous inflammation. CAA with these features was distinguished from perforation or abscess formation because different antibiotic regimens were required. The SOP was the subject of presentations at institutional journal clubs throughout 2020 and was made available on the clinic’s intranet for permanent access. Contact with the ABS team as well as the departments of clinical microbiology, infectious disease, and pharmacy was provided for any questions that arose.

To investigate surgeons’ compliance and detect differences in outcomes, we performed a single-center retrospective analysis of all children (0–15 years) who underwent appendectomy coded according to ICD-10 (K35.XX) between 2018 and 2022. Patients were assigned to either “before” (2018/2019) or “after” (2021/2022) the introduction of this ABS-compliant peri-/postoperative antibiotic regimen in the summer of 2020. The entire year 2020 was considered a transition period and therefore excluded from the final comparison. Of the total of 435 patients treated for appendicitis in 2018, 2019, 2021, and 2022, 49 were excluded from the study because they were miscoded as AA, were treated conservatively, or underwent elective appendectomy for another reason (e.g., interval appendectomy after acute inflammation). Of the remaining 386 cases, digital and/or analog charts, surgical documentation, as well as microbiological and histological findings were reviewed to evaluate defined end points (length of stay [LOS], complication rate, adequate implementation and duration of postoperative antibiotic therapy, correct antibiotic agent used, adequate and on time de-escalation and oralization, length of antibiotic therapy [LAT], and appropriate escalation of antibiotic therapy if indicated).

**Figure 2:**
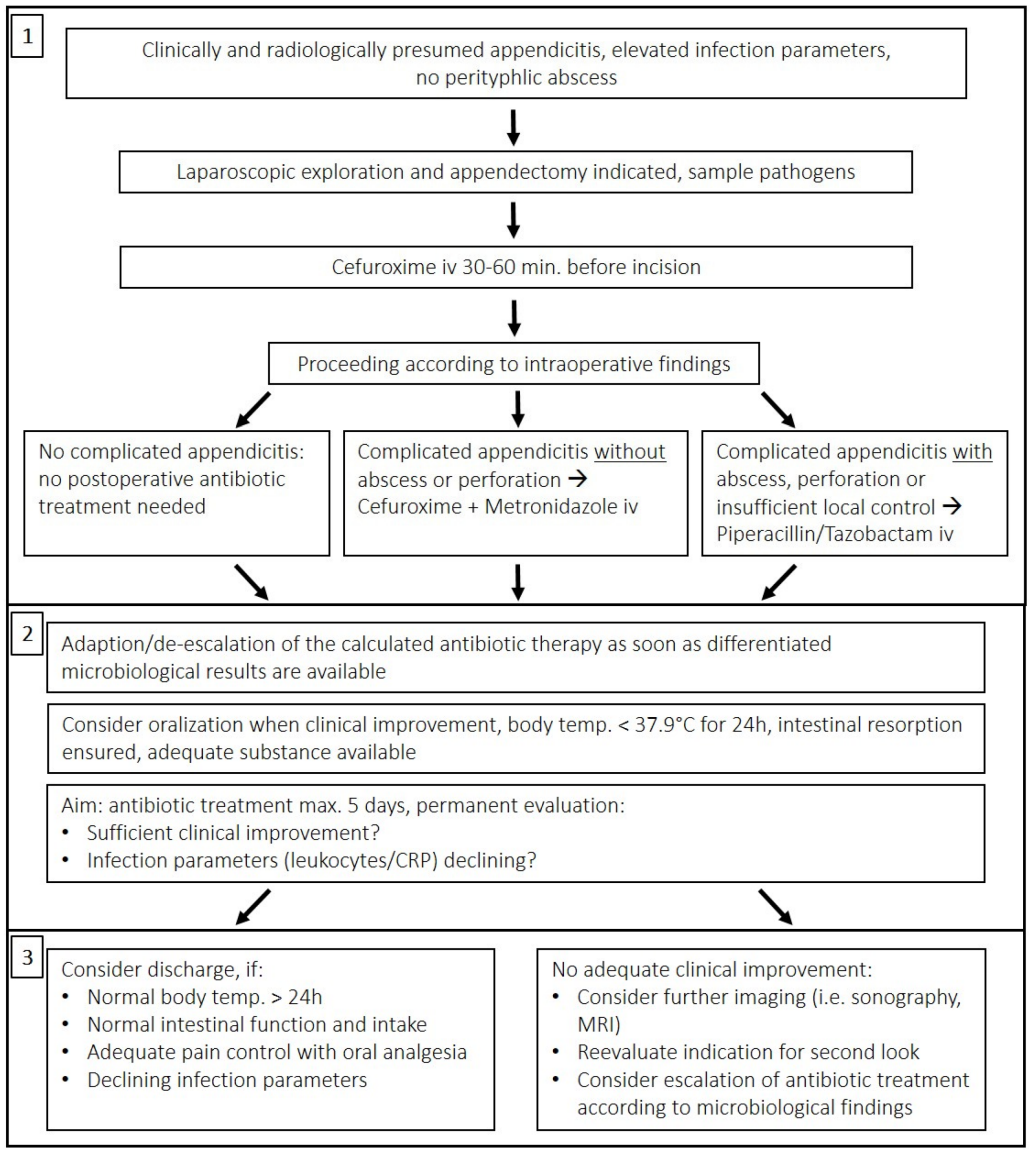
Postoperative management after appendectomy, with consideration of intraoperative findings and frequent clinical evaluation according to scientific evidence and good clinical practice

Our algorithm depicting the recommended treatment after appendectomy was developed using the implemented SOP, current scientific evidence [8], [21], [22], [23], [24], [25], [26], [27], [28], [29], and good clinical practice (GCP) as reviewed by the heads of the departments of pediatric infectiology, pharmacology, clinical microbiology, and pediatric surgery (Figure 2 (Fig. 2)).

For evaluation, we developed a scoring system that awarded points for correct decision-making at key branching points following the algorithm. These marked either a significant change in patient status (e.g., surgery, discharge) or findings that required a specific action (based on the intraoperative macroscopic aspect, postoperative clinical course, laboratory or microbiological parameters). A maximum of 20 points (=100% quality of antibiotic therapy [QOT]) was awarded for correct therapeutic decisions throughout the course. In addition, points were deducted for serious deviations from the key principles of ABS. Patients’ postoperative antibiotic regimens were evaluated individually and independently by two reviewers who were considered experienced in antimicrobial treatment and ABS and who, in most cases, were not involved in the initial surgical decision. A discrepancy of more than 2 points (10%) within the independent assessment required comparison and mutual reconsideration. For reviewers and interested readers, we provide additional information on the structure and approach of this assessment in Attachment 1).

Statistical analysis was performed using Excel (version 16.8) and Graphpad Prism (version 10). The 2018/2019 and 2021/2022 groups largely reflect a similar distribution in terms of disease severity and complication and conversion rates, as shown in Table 1 (Tab. 1). Therefore, we consider the groups to be essentially comparable.

A Gaussian distribution was tested and excluded in all samples with regular normality tests (Shapiro-Wilk, Kolmogorov-Smirnov, QQ-Plot). Therefore, statistical significance was tested using the Mann-Whitney test, with p-values <0.05 defined as statistically significant. Bars show mean and standard error; box-and-whisker plots show median and 95% confidence interval.

## Results

In our central European tertiary care pediatric surgery unit, we performed an average of 97 laparoscopic appendectomies per year between 2018 and 2022. 40–50% of the patients included here presented with advanced stage appendicitis (CAA), with approximately ¼ already macroscopically perforated (PAA) or presenting with intra-abdominal abscess (IAA) at the time of surgery (Table 1 (Tab. 1)). A chi-squared test of the means of 2018/2019 vs. 2021/2022 showed no significant difference in the distribution of disease severity, conversion rate, and complication rate (p-value 0.9108).

**Figure 3:**
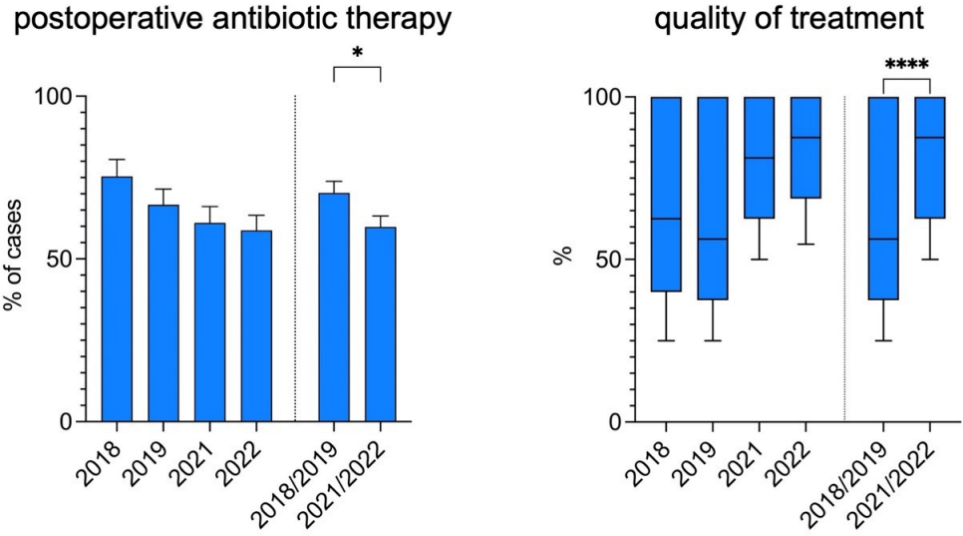
Percentage of cases receiving postoperative antibiotic treatment after appendectomy and quality of the antibiotic treatment before and after the implementation of a standardized antibiotic regimen

From 2018/2019 to 2021/2022, the number of cases in which postoperative antibiotic therapy was administered decreased by 10.5%, from 70.3% to 59.8% (p-value 0.036). This was primarily due to a decrease in postoperative therapy in the NCA group from 50.5% of cases in 2018/19 to 34.2% of cases in 2021/22, while the indication for postoperative antibiotic therapy in cases of advanced stage appendicitis (CAA/PAA/IAA) showed a stable proportion of 91.9% in 2018/19 and 88.2% in 2021/22 (Figure 3 (Fig. 3)).

The quality of this antibiotic treatment in terms of the principles of ABS and GCP was significantly improved. While the median overall quality of antibiotic therapy (QOT) was 56.3% in 2018/2019, it reached 87.5% in 2021/2022 (p-value <0.0001) with an upward trend over time correlating with the therapists’ increasing compliance with following the protocol.

**Figure 4:**
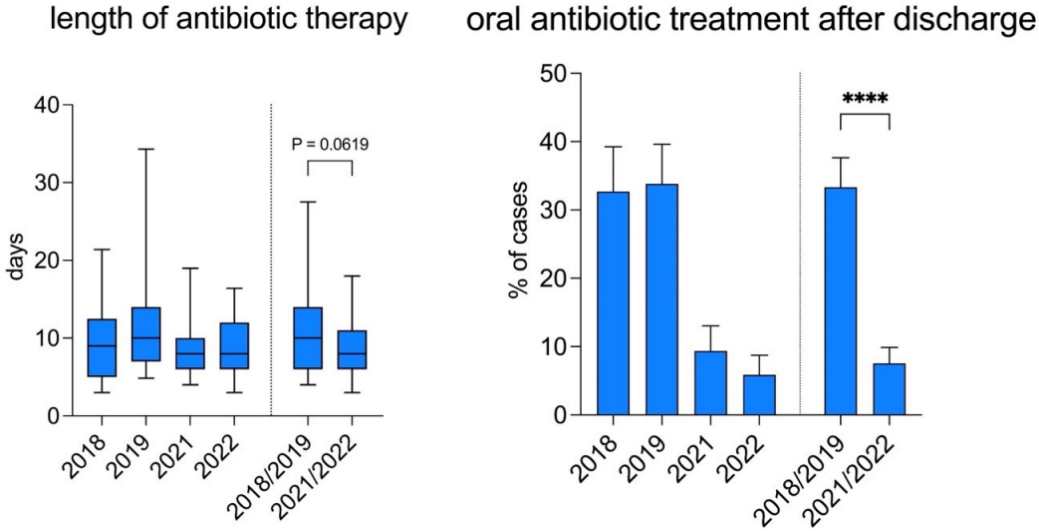
Duration of antibiotic treatment after appendectomy for complicated appendicitis and percentage of cases with (prolonged) oral antibiotic treatment after discharge before and after implementation of a standardized antibiotic regimen

The median length of the antibiotic therapy (LAT) after appendectomy for advanced stage appendicitis was also reduced by 2 days overall (10 days in 2018/2019 vs. 8 days in 2021/2022, p-value 0.062). In addition, the mean rate of (extended) oral antibiotic treatment after discharge was lowered from 33.3% in 2018/2019 to 7.6% in 2021/2022 (p-value <0.0001) (Figure 4 (Fig. 4)). 

There was no relevant change in overall median length of the hospital stay (LOS) after appendectomy (5 days 2018/2019 vs. 5 days 2021/2022, p-value 0.2208) or in the number of primary postoperative complications, such as SSI or secondary intra-abdominal abscess formation (Table 1 (Tab. 1)).

## Discussion

SOPs improve the effectiveness of ABS programs and the appropriateness of antibiotic regimens [30], [31]. However, although there are occasional reports of improved outcomes and minimization of postoperative complications with the implementation of an SOP after appendectomy in pediatric surgery, robust data remain scarce [32], [33], [34]. This is largely due to the fact that studies in pediatric surgery have primarily focused on the comparison of drug regimens within individual stages of AA [35], [36], [37]. Also, a clear differentiation of the intraoperative macroscopic findings, especially what objectively defines complicated acute appendicitis, has not yet been found. Therefore, a valid comparison of these studies is difficult [7], [38]. In addition, health care resources and structures vary widely between regions of the world, making it even more difficult to compare scientific findings and convert them into local GCP. As a result, common recommendations are based on limited evidence and are often at odds with common practice [39]. Thus, inter-surgeon agreement is poor with respect to subjective appendicitis classification and objective utilization of postoperative antibiotics [40]. In Europe, for example, therapeutic approaches vary widely from one institution to another [41]. To our knowledge, for these and other reasons, there is no scientifically proven, published SOP that condenses an optimal approach defining and reflecting all stages of AA in the context of healthcare resources in Europe. 

The aim of this study was to evaluate how the implementation of an SOP, considering intraoperative findings, local microbial conditions and GCP, influences the QOT for pediatric patients with AA of all stages after primary LA. The underlying algorithm was developed with respect to existing published protocols and previous literature with the goal of finding the optimal therapy to prevent SSI or postoperative abscess formation, shorten the length of hospital stays and antibiotic treatment as responsibly as possible, and cover the expected spectrum of pathogens with the initially calculated antibiotic regimen (see also Attachment 1) [16], [35], [37], [42], [43], [44], [45], [46], [47]. 

First, this analysis shows that the implementation of standardized ABS-compliant postoperative antibiotic treatment based on intraoperative findings and surgeon’s assessment in cases of acute appendicitis was associated with improvements in the postoperative care of affected children in terms of quality and duration of antibiotic therapy. This did not increase the incidence of primary complications or prolong inpatient treatment to any relevant extent. 

Second, to assess quality of treatment, we introduced an algorithm for retrospective evaluation of current institutional practice versus “ideal care”, as shown in Figure 2 (Fig. 2). This approach may be a useful tool that could be adapted and then potentially used for evaluation in other circumstances to assess ABS compliance under numerous typical surgical circumstances requiring anti-infective treatment. Consequently, it may help to further implement the principles of ABS in pediatric surgery.

## Limitations

The algorithm presented here is based on the current evidence for antibiotic therapy after laparoscopic appendectomy given different stages of acute appendicitis and the microbial distribution as reviewed in the contemporary literature, with the limitations mentioned above. Further research is needed to elucidate the evidence for antimicrobial therapy in pediatric acute appendicitis.

Although we were able to show that standardization of postoperative therapy is associated with a reduction in the duration of such therapy, the decision of when to discharge and when to discontinue antibiotic therapy is typically influenced not only by objective criteria, but also by the experience, understanding of safety, and personal attitude of the surgeon in charge, who in turn is influenced by his or her training as well as the “local culture” in a specific clinical setting. This aspect must be considered in the interpretation of the present results and in the design of further studies, as it may be a source of bias. For instance, the effect of implementing objective discharge criteria, as shown in Figure 2 (Fig. 2) (Section 3), on treatment efficacy and outcome may be of interest for further investigation.

Lastly, this study has the inherent limitations of a single-center retrospective analysis. Therefore, further prospective multicenter studies are needed.

## Conclusion

The implementation of SOP for peri- and postoperative care after appendectomy, considering the principles of ABS, was associated with an improvement in the quality of postoperative antibiotic treatment in pediatric patients, especially in cases of advanced stage appendicitis – as summarized in the graphical abstract. This was demonstrated using a novel algorithm to evaluate the current QOT compared to the standards of ABS in acute appendicitis. In general, ABS models should be better adapted to pediatric surgical procedures in order to develop acceptable implementation strategies that further optimize antibiotic prescribing in pediatric surgery.

## Notes

### Conflict of interest

The authors declare that they have no competing interests.

### Further acknowledgments

None.

### Funding

No external financial support has been provided.

## Supplementary Material

Additional information on the structure and approach of the assessment

## Figures and Tables

**Table 1:**
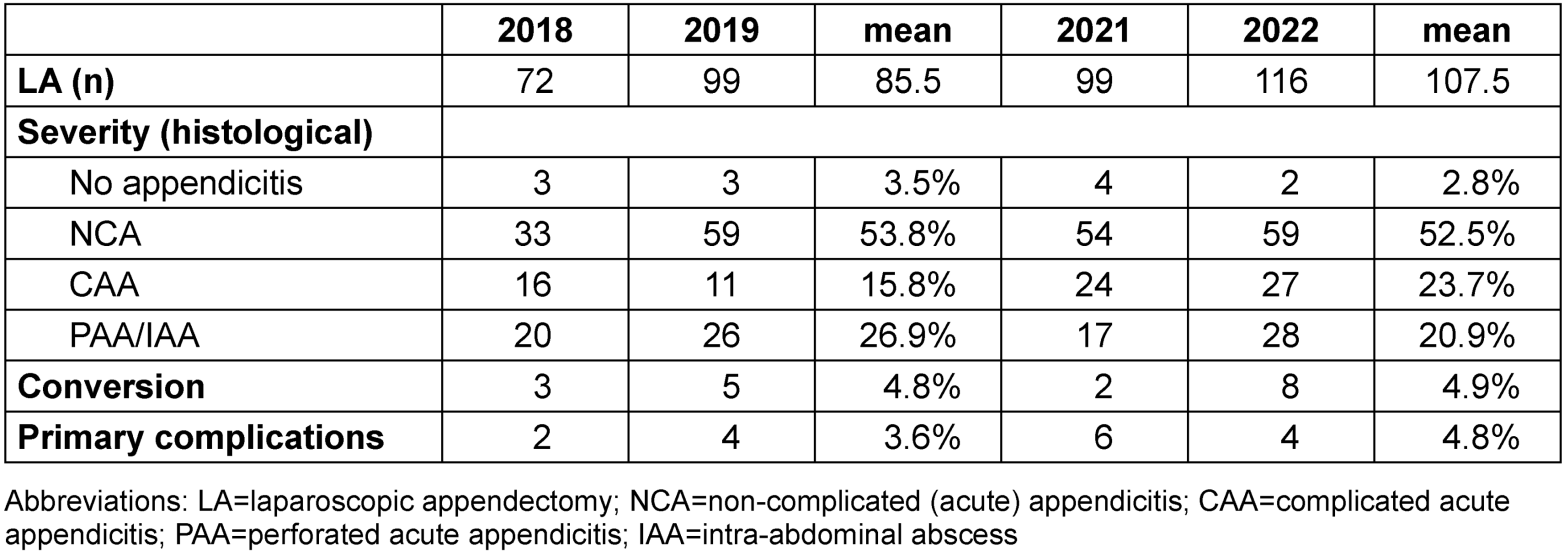
Severity of acute appendicitis as macroscopically described at the time of surgery, conversion, and primary complications before (2018/2019) and after (2021/2022) implementation of a standard operating procedure (SOP) in 2020
