# Supplementary material for: Better care for children with appendicitis: implementation of antibiotic stewardship optimizes postoperative therapy
Source: GMS Hyg Infect Control. 2025 Mar 5;20:Doc06. doi: 10.3205/dgkh000535 (PMC12059807; doi:10.3205/dgkh000535)
Supplement: Additional information on the structure and approach of the assessment [file HIC-20-06-s-001.pdf]

Attachment

To objectively evaluate the correctness of therapeutic decisions regarding antibiotic therapy during the peri-/postoperative course of children undergoing appendectomy for acute appendicitis, each patient's course was compared with the algorithm in Figure 1. ABS-conform decisions at key time points during the course were scored as shown in supplementary Table 1. Each investigator awarded a maximum of 10 points. In addition, a maximum of 1 penalty point per section and reviewer was distributed for serious deviations from the main principles of ABS. Consequently, a maximum of 20 points were given for correct therapeutic decision making throughout the course of peri-/postoperative antibiotic treatment. Thus, the quality of antibiotic therapy (QOT) was graduated as a percentage of the maximum 20 possible points. A discrepancy of more than 2 points (10%) within the independent assessment required comparison and mutual reconsideration. If a section was not applicable, the maximum possible points were minimized accordingly (e.g. patient with uncomplicated appendicitis, no adjustment for insufficient clinical improvement necessary, max. 8 points per examiner=100%).

For example, patient 'XY' was indicated for laparoscopic exploration due to clinical, sonographic and laboratory indicators of complicated acute appendicitis (CAA). Cefuroxime was administered on time (1 pt), intraoperative findings confirmed the diagnosis of perforated acute appencitis (PAA), and putrid fluid was collected (1 pt). Postoperatively, antibiotic therapy with Cefuroxime and Metronidazole was started (1 pt for indication of antibiotic therapy and 1 penalty pt for insufficient choice of regimen). The microbiological examination revealed a resistant Enterococcus subspecies intra-abdominally. Based on this finding, the antibiotic therapy was sufficiently escalated to Piperacillin/Tazobactam on day 2, taking adequately into account the pathogen/resistance (2 points for appropriate adjustment). Finally, the patient was discharged in good clinical condition after 9 days of intravenous antibiotic therapy (4 pts). In summary, the evaluator awarded 8 out of 10 possible points, which corresponds to 80% QOT.

Supplementary Table 1: Basis for the independent evaluation of the peri-/postoperative antibiotic therapy after pediatric appendectomy

Section	Goal	Points	Penalty-points
**1**	Adequate indication for (laparoscopic) exploration/appendectomy and abdominal fluid collection	1	1
	On time perioperative administration of Cefuroxime	1	
	Comprehensible indication of the (calculated) postoperative antibiotic regimen due to intraoperative findings (1st pt.) and correct application (2nd pt.)	2	
**2**	Continuation/adaption/de-escalation of the antibiotic therapy due to determined pathogens (1st pt.) and reasonable application (2nd pt.)	2	1
	Oralization considered and established reasonably	1	
	Avoidance of a prolonged antibiotic therapy	1	
**3**	Adequate reaction to insufficient clinical improvement: Escalation considering pathogens/resistance (1st pt.) and reasonable application (2nd pt.)	2	1
**Max. points per examiner**		**10**	**3**

Attachment to: Beltz S, Fischer S, Huenger F, Vahdad R, Kalhoff H, Leutner A. Better care for children with appendicitis: implementation of antibiotic stewardship optimizes postoperative therapy. GMS Hyg Infect Control. 2024;19:Doc06. DOI: 10.3205/dgkh000535
